# Supplementary material for: Langerhans cells shape postnatal oral homeostasis in a mechanical-force-dependent but microbiota and IL17-independent manner
Source: Nat Commun. 2023 Sep 12;14:5628. doi: 10.1038/s41467-023-41409-0 (PMC10497507; doi:10.1038/s41467-023-41409-0)
Supplement: Supplementary file 1 — Supplementary Information [file 41467_2023_41409_MOESM1_ESM.pdf]

Supplementary Information

Langerhans cells shape postnatal oral homeostasis in a mechanical-force-dependent but microbiota and IL17-independent manner

Yasmin Jaber, Yasmine Netanely, Reem Naamneh, Or Saar, Khaled Zubeidat, Yasmin Saba, Olga Georgiev, Paz Kles, Or Barel, Yael Horev, Omri Yosef, Luba Eli-Berchoer, Chen Nadler, Gili Betser-Cohen, Hagit Shapiro, Eran Elinav, Asaf Wilensky, and Avi-Hai Hovav

Supplementary Figure

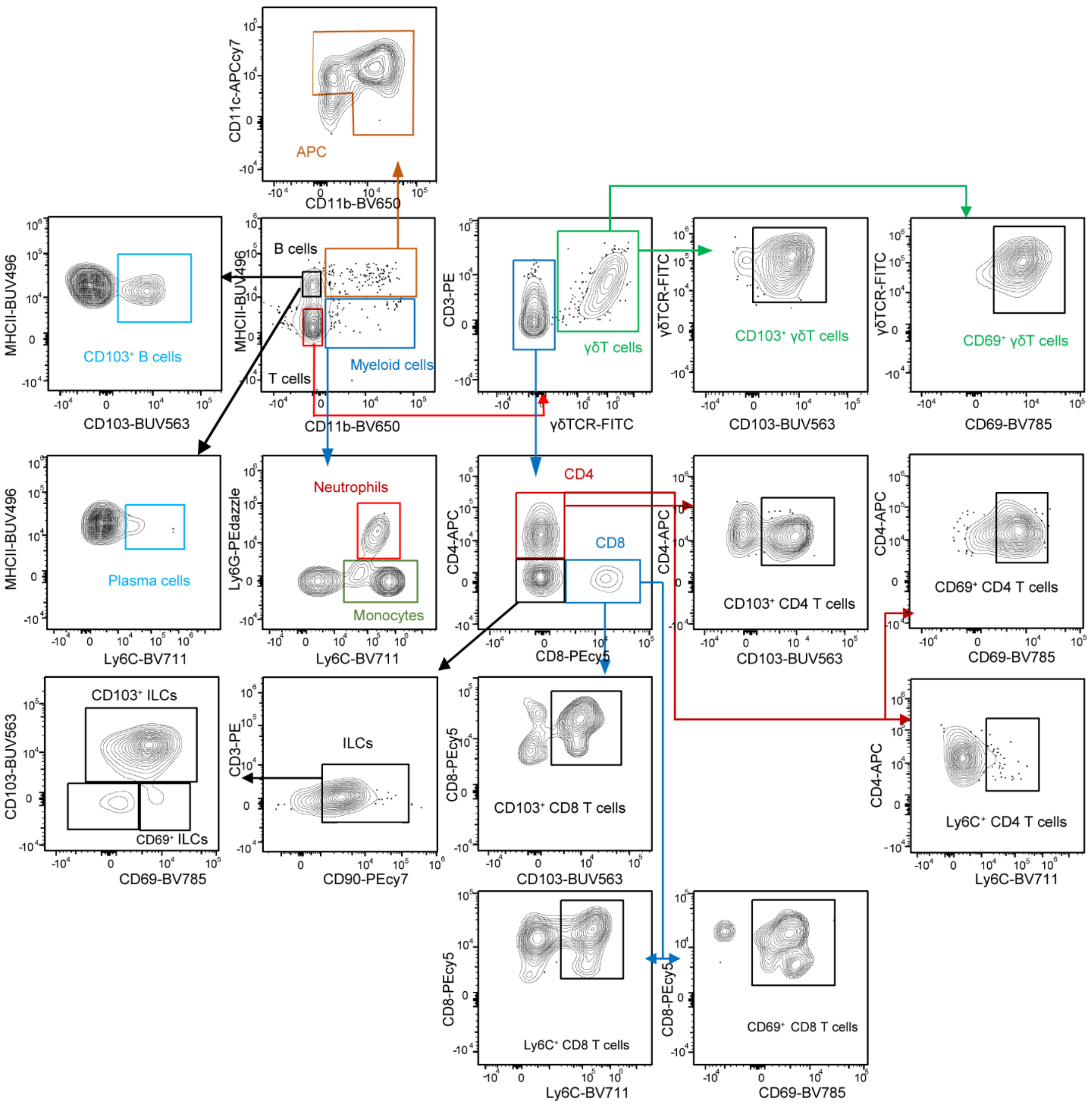

Fig. S1: Gating strategy for flow cytometry analysis of oral tissues

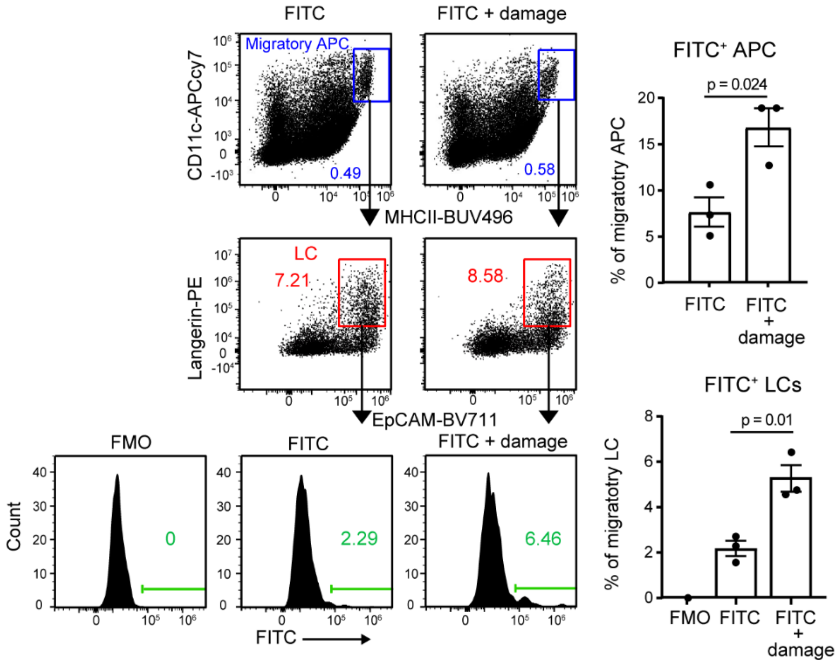

Fig. S2: Mechanical damage induces migration of gingival LCs to the cervical LNs.

Adult B6 mice were painted with FITC solution, a few hours later, a mechanical force was applied to the gingiva, and 24 hr later the cervical LNs were collected for flow cytometry analysis. Representative FACS plots and graphs show the mean frequencies + SEM (n=3) of FITC-positive APCs and LCs in the cervical LNs. Representative data of one out of two independent experiments. *p*-value from a two-tailed, unpaired t-test using GraphPad Prism.

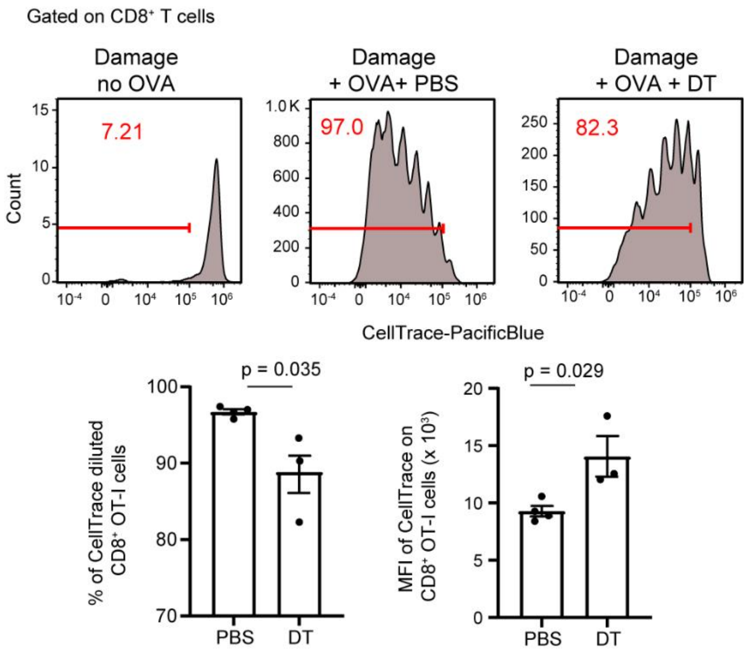

Fig. S3: LCs present antigen to T cells upon application of mechanical damage

Langerin-DTR mice were injected with DT or PBS and 24hr later the mice were adoptively transferred with 5×10^6 CellTrace-labeled solenocytes purified from OT-I mice. Mechanical damage was then induced in the oral epithelium using a cotton swab soaked with the antigen ovalbumin. Three days later the cervical LNs were collected for analysis. Representative flow cytometry plots and graphs present the mean dilution + SEM of the CellTrace labeling and its mean fluorescence intensity (MFI) + SEM on the proliferating OT-I $CD8^+$ T cells (n=3). Representative data of one out of two independent experiments. *p*-value from a two-tailed, unpaired t-test using GraphPad Prism.

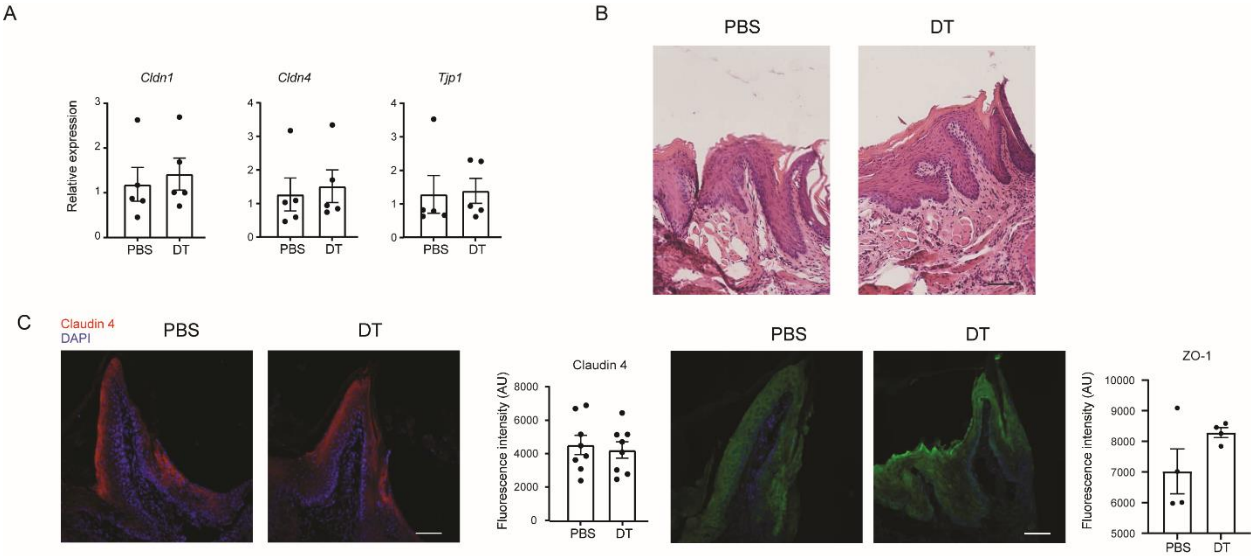

Fig. S4: Depletion of LC during weaning does not affect the expression of tight junction-associated proteins.

LCs were depleted in langerin-DTR during weaning and analyzed at the eighth week of life. (A) Relative expression of the note genes in the gingiva of mice that were either depleted of LCs (DT) or not (PBS). Graphs present the transcript levels quantified by RT-PCR and normalized to PBS-treated mice depicted as the mean + SEM (n=5 mice). Representative data from two independent experiments. (B) Representative hematoxylin and eosin (H&E) histological cross-sections were prepared from the gingiva DT and PBS-treated mice. Scale bar, 50 mm. (C) Immunofluorescence of gingival cross-sections of DT and PBS-treated mice with antibodies directed against claudin 4 (red), ZO-1 (green), and DAPI (blue). Representative images and graphs show the staining intensity as mean values + SEM (n=8 for claudin, n=4 for ZO-1). Representative data from two independent experiments. Scale bar, 50 mm.

Supplementary Tables

Table 1: List of primers used in this study.

**Gene**	**Forward**	**Reverse**
Gapdh	AGTTGGGATAGGGCCTCTCTT	TCCCACTCTTCCACCTTCGA
Ifng	TCAAGTGGCATAGATGTGGAAGAA	TGGCTCTGCAGGATTTTCATG
Tnfa	CCTGTAGCCCACGTCGTAG	GGGAGTAGACAAGGTACAACCC
Foxp3	AGCAGTGTGGACCGTAGATGA	GGCAGGGATTGGAGCACTT
Il17a	TCAGCGTGTCCAAACACTGAG	CGCCAAGGGAGTTAAAGACTT
16s	AGAGTTTGATCCTGGCTC	TGCTGCCTCCCGTAGGA GT
Ccl20	CCAAGGGCTGCAAGAGAACT	TGAGTTGGACCGTGAACCAC
Cxcl1	ATCCAGAGCTTGAAGGTGTTG	GTCTGTCTTCTTTCTCCGTTACTT
Cxcl2	TGAACAAAGGCAAGGCTAACT	CAGGTACGATCCAGGCTTCC
Ccl2	TGCTGACCCCAAGAAGGAAT	TTTGGTTCCGATCCAGGTTTT
Cldn1	GGGGACAACATCGTGACCG	AGGAGTCGAAGACTTTGCACT
Cldn4	GTCCTGGGAATCTCCTTGGC	TCTGTGCCGTGACGATGTTG
Tjp1	GCTTTAGCGAACAGAAGGAGC	TTCATTTTTCCGAGACTTCACCA
