# Supplementary material for: Langerhans cells shape postnatal oral homeostasis in a mechanical-force-dependent but microbiota and IL17-independent manner
Source: Nat Commun. 2023 Sep 12;14:5628. doi: 10.1038/s41467-023-41409-0 (PMC10497507; doi:10.1038/s41467-023-41409-0)
Supplement: Supplementary file 3 — Reporting Summary [file 41467_2023_41409_MOESM3_ESM.pdf]

nature portfolio

Corresponding author(s): Avi-Hai Hovav

Last updated by author(s): Jul 17, 2023

# Reporting Summary

Nature Portfolio wishes to improve the reproducibility of the work that we publish. This form provides structure for consistency and transparency in reporting. For further information on Nature Portfolio policies, see our Editorial Policies and the Editorial Policy Checklist.

## Statistics

For all statistical analyses, confirm that the following items are present in the figure legend, table legend, main text, or Methods section.

| n/a | Confirmed | |
|---|---|---|
| ☐ | ☒ | The exact sample size (*n*) for each experimental group/condition, given as a discrete number and unit of measurement |
| ☐ | ☒ | A statement on whether measurements were taken from distinct samples or whether the same sample was measured repeatedly |
| ☐ | ☒ | The statistical test(s) used AND whether they are one- or two-sided<br>*Only common tests should be described solely by name; describe more complex techniques in the Methods section.* |
| ☐ | ☒ | A description of all covariates tested |
| ☐ | ☒ | A description of any assumptions or corrections, such as tests of normality and adjustment for multiple comparisons |
| ☐ | ☒ | A full description of the statistical parameters including central tendency (e.g. means) or other basic estimates (e.g. regression coefficient) AND variation (e.g. standard deviation) or associated estimates of uncertainty (e.g. confidence intervals) |
| ☐ | ☒ | For null hypothesis testing, the test statistic (e.g. *F*, *t*, *r*) with confidence intervals, effect sizes, degrees of freedom and *P* value noted<br>*Give P values as exact values whenever suitable.* |
| ☐ | ☒ | For Bayesian analysis, information on the choice of priors and Markov chain Monte Carlo settings |
| ☐ | ☒ | For hierarchical and complex designs, identification of the appropriate level for tests and full reporting of outcomes |
| ☒ | ☐ | Estimates of effect sizes (e.g. Cohen's *d*, Pearson's *r*), indicating how they were calculated |

*Our web collection on statistics for biologists contains articles on many of the points above.*

## Software and code

Policy information about availability of computer code

Data collection

"RNA ScreenTape kit (catalog #5067-5576; Agilent Technologies, Santa Clara, CA), D1000 ScreenTape kit (catalog #5067-5582; Agilent Technologies), Qubit® RNA HS Assay kit (catalog # Q32852; Invitrogen, Carlsbad, CA) and Qubit® DNA HS Assay kit (catalog #32854; Invitrogen) were used for each specific step purpose for quality control of RNA and DNA libraries. For mRNA library preparation: KAPA Stranded mRNA-Seq Kit with mRNA Capture Beads (kapabiosystems, KK8421, https://www.kapabiosystems.com/) was used. In brief, 1ug was used for the library construction; library was eluted in 20 ul of elution buffer. All DNA samples libraries were adjusted to 4 nM, then pooled in one tube.
Multiplex samples Pool (1.6 pM including PhiX 1%) was loaded in NextSeq 500/550 High Output v2 kit (75 cycles) cartridge (catalog #FC-404-1005; Illumina, San Diego, CA) and loaded on NextSeq 500 System machine (Illumina), with 75 cycles and single-Read Sequencing conditions."

| | |
|---|---|
| Data analysis | Raw reads were processed according to the QuantSeq User Guide recommendations, reads were trimmed at their 5' end to remove the first 12 bases, then low quality and technical bases were removed from the 3' end using cutadapt (version 1.12) (3). Finally, low quality reads, with more than 30 percent of the bases with quality below 20, were filtered out using the FASTX package (version 0.0.14). Processed reads were aligned against the mouse genome using TopHat (v2.1.1) (4). The genome version was GRCm38, with annotations from Ensembl release 89. Htseq-count (version 0.6.0) (5) was then used for quantification of raw counts per gene per sample, excluding short or otherwise unwanted gene types, such as rRNA or miRNA. Normalization and differential expression analysis were performed with the DESeq2 package (version 1.12.4). Genes with a sum of counts less than 10 over all samples were filtered out prior to normalization. Differential expression, comparing 8-weeks to 1-week old mice, was calculated with default parameters, except not using the independent filtering algorithm. Statistical significance 6 threshold was taken as adjusted p-value (padj) less than 0.1. Exact commands with the full parameters used can be found under GEO accession. |

For manuscripts utilizing custom algorithms or software that are central to the research but not yet described in published literature, software must be made available to editors and reviewers. We strongly encourage code deposition in a community repository (e.g. GitHub). See the Nature Portfolio guidelines for submitting code & software for further information.

## Data

Policy information about availability of data

All manuscripts must include a data availability statement. This statement should provide the following information, where applicable:

- Accession codes, unique identifiers, or web links for publicly available datasets
- A description of any restrictions on data availability
- For clinical datasets or third party data, please ensure that the statement adheres to our policy

The RNA-seq data generated in this study have been deposited in the NCBI Gene Expression Omnibus GEO database under accession code GSE232790. GSEA was run against the hallmark gene set collection from the molecular signature database (MSigDB, v6.2, July 2018). All data are available in the main text and the supplementary materials.

## Human research participants

Policy information about studies involving human research participants and Sex and Gender in Research.

| | |
|---|---|
| Reporting on sex and gender | N/A |
| Population characteristics | N/A |
| Recruitment | N/A |
| Ethics oversight | N/A |

Note that full information on the approval of the study protocol must also be provided in the manuscript.

# Field-specific reporting

Please select the one below that is the best fit for your research. If you are not sure, read the appropriate sections before making your selection.

☒ Life sciences ☐ Behavioural & social sciences ☐ Ecological, evolutionary & environmental sciences

For a reference copy of the document with all sections, see nature.com/documents/nr-reporting-summary-flat.pdf

# Life sciences study design

All studies must disclose on these points even when the disclosure is negative.

| | |
|---|---|
| Sample size | Common samples sizes of n=5 with a minimum of n=3 was chosen based on previous publications in the field. (Koren et al., 2021, Pfister et al. 2020) |
| Data exclusions | No data were excluded from analysis. |
| Replication | All experiments were preformed in this manuscript at least two times independent biological replicates. All attempt to reproduce the results were successful. |
| Randomization | Age and sex matched mice were assigned randomly to each experimental and control group. |
| Blinding | In the RNAseq and taxonomic analyses, the experiments were blinded as the analysis was conducted by specialists who did not know the identity of the samples. In experiments involving transgenic mice or the induction of damage, the experiments were not blinded as the mice were cohoused and thus must be marked to enable identification. In experiments involving time course analysis of germ-free mice, blinding was impossible due to differences in the maturity (e.g., body size) and the different housing conditions (GF mice). |

# Reporting for specific materials, systems and methods

We require information from authors about some types of materials, experimental systems and methods used in many studies. Here, indicate whether each material, system or method listed is relevant to your study. If you are not sure if a list item applies to your research, read the appropriate section before selecting a response.

## Materials & experimental systems

| n/a | Involved in the study |
| --- | --- |
| ☐ | ☒ Antibodies |
| ☒ | ☐ Eukaryotic cell lines |
| ☒ | ☐ Palaeontology and archaeology |
| ☐ | ☒ Animals and other organisms |
| ☒ | ☐ Clinical data |
| ☒ | ☐ Dual use research of concern |

## Methods

| n/a | Involved in the study |
| --- | --- |
| ☒ | ☐ ChIP-seq |
| ☐ | ☒ Flow cytometry |
| ☒ | ☐ MRI-based neuroimaging |

## Antibodies

Antibodies used

Rat PE anti-mouse CD3 BioLegend Cat# 100206; RRID: AB_312663, Clone: 17A2
Armenian hamster FITC anti-mouse TCR γ/δ BioLegend Cat# 118106; RRID: AB_313830, Clone: GL3
Rat Brilliant Violet 421 anti-mouse IFN-γ BioLegend Cat# 505829; RRID: AB_10897937, Clone: XMG1.2
Mouse Pacific Blue anti-mouse CD45.2 BioLegend Cat# 109820; RRID: AB_492872, Clone: 104
Rat APC anti-mouse IL17-A BioLegend Cat# 506915; RRID: AB_536017, Clone: TC11-18H10.1
Mouse PE anti-mouse, human Langerin (CD207) BioLegend Cat# 144204; RRID: AB_2561499, Clone: 4C7
Armenian hamster Brilliant Violet 605 anti-mouse TCR β chain BioLegend Cat# 109241; RRID: AB_2629563, Clone: H57-597
Rat PerCP/Cyanine 5.5 anti-mouse I-A/I-E BioLegend Cat# 107626; RRID: AB_ 2191071, Clone: M5/114.15.2
Rat APC anti-mouse Ly-6G BioLegend Cat# 127614; RRID: AB_2227348, Clone: 1A8
Rat Brilliant Violet 711 anti-mouse CD326 (EpCAM) BioLegend Cat# 118233; RRID: AB_2632775, Clone: G8.8
Rat PE/Cyanine7 anti-mouse Ly-6C BioLegend Cat# 128018; RRID: AB_1732082, Clone: HK1.4
Armenian hamster Brilliant Violet anti-mouse CD69 BioLegend Cat# 104543; RRID: AB_2629640, Clone: H1.2F3
CLDN4 / Claudin 4 Rabbit anti-Human Antibody LSBio Cat# LS-B14814, Clone: ABT-CLD4
Goat Anti-Mouse IgG H&L (HRP) abcam Cat# ab6789; RRID: AB_955439, Clone: Polyclonal
Goat Anti-Mouse IgA alpha chain (HRP) abcam Cat# ab97235; RRID: AB_ 10681186, Clone: Polyclonal
Anti-GAPDH antibody [EPR16891] - Loading Control abcam Cat# ab181602; RRID: AB_ 2630358, Clone: EPR16891
CD207 / Langerin Rabbit anti-Mouse Polyclonal (aa26-44) Antibody LSBio Cat# LS-C735, Clone: aa26-44
Rat PE/Cyanine7 anti-mouse/human CD45R/B220 BioLegend Cat# 103222; RRID: AB_ 313005, Clone: RA3-6B2
Rat Brilliant Violet 711™ anti-mouse Ly-6C BioLegend Cat# 128037; RRID: AB_ 2562630, Clone: HK1.4
Rat PE/Cyanine7 anti-mouse/human CD11b BioLegend Cat# 101216; RRID: AB_312799, Clone: M1/70
Rat APC/Cy7 anti-mouse CD4 BioLegend Cat# 100414; RRID: AB_ 312699, Clone: GK1.5
Rat PE/Cyanine5 anti-mouse CD8a BioLegend Cat# 100709; RRID: AB_312749, Clone: 53-6.7
Rat Brilliant Violet 650TM anti-mouse/human CD11b BioLegend Cat# 101259; RRID: AB_ 2566568 Clone: M1/70
Rat PE/DazzleTM 594 anti-mouse Ly-6G BioLegend Cat# 127647; RRID: AB_ 2566318, Clone: 1A8
Armenian hamster APC/Cyanine7 anti-mouse CD11c BioLegend Cat# 117324; RRID: AB_ 830649, Clone: N418
Rat anti-Mouse I-A/I-E BUV496 M5/114.15.2 BD Biosciences Cat# 750281; RRID: AB_ 2874472, Clone: M5/114
Rat BD741261 BUV563 anti-mouse CD103 BD Biosciences Cat# 741261; RRID: AB_ 2870808, Clone: M290
Rat Alexa Fluor® 488 anti-mouse FOXP3 BioLegend Cat# 126406; RRID: AB_ 1089113, Clone: MF-14

Validation

Similar results with validation results in relevant citations:
Rat PE anti-mouse CD3 BioLegend Cat# 100206; RRID: AB_312663 - Zubeidat K. et al. Cell Reports (2023)
Armenian hamster FITC anti-mouse TCR γ/δ BioLegend Cat# 118106; RRID: AB_313830 - Koren N. et al. Cell Host & Microbe (2021)
Rat Brilliant Violet 421 anti-mouse IFN-γ BioLegend Cat# 505829; RRID: AB_10897937 - Wilharm A. et al. PNAS (2019)
Mouse Pacific Blue anti-mouse CD45.2 BioLegend Cat# 109820; RRID: AB_492872 - Barel O. et al. JDR (2022)
Rat APC anti-mouse IL17-A BioLegend Cat# 506915; RRID: AB_536017 - Wilharm A. et al. PNAS (2019)
Mouse PE anti-mouse, human Langerin (CD207) BioLegend Cat# 144204; RRID: AB_2561499 - Saba Y. et al. PNAS (2022)
Armenian hamster Brilliant Violet 605 anti-mouse TCR β chain BioLegend Cat# 109241; RRID: AB_2629563 - Horev Y. et al. Mucosal Immunology (2020)
Rat PerCP/Cyanine 5.5 anti-mouse I-A/I-E BioLegend Cat# 107626; RRID: AB_ 2191071 - Barel O. et al. JDR (2022)
Rat APC anti-mouse Ly-6G BioLegend Cat# 127614; RRID: AB_2227348 - Wald S. et al. JDR (2021)
Rat Brilliant Violet 711 anti-mouse CD326 (EpCAM) BioLegend Cat# 118233; RRID: AB_2632775 - Saba Y. et al. PNAS (2022)
Rat PE/Cyanine7 anti-mouse Ly-6C BioLegend Cat# 128018; RRID: AB_1732082 - Zubeidat K. et al. Cell Reports (2023)
Armenian hamster Brilliant Violet anti-mouse CD69 BioLegend Cat# 104543; RRID: AB_2629640 - Zubeidat K. et al. Cell Reports (2023)
CLDN4 / Claudin 4 Rabbit anti-Human Antibody LSBio Cat# LS-B14814 Goat Anti-Mouse IgG H&L (HRP) abcam Cat# ab6789; RRID: AB_955439 - Koren N. et al. Cell Host & Microbe (2021)
Goat Anti-Mouse IgA alpha chain (HRP) abcam Cat# ab97235; RRID: AB_ 10681186 - Zubeidat K. et al. Cell Reports (2023)
Anti-GAPDH antibody [EPR16891] - Loading Control abcam Cat# ab181602; RRID: AB_ 2630358 - Zubeidat K. et al. Cell Reports (2023)
CD207 / Langerin Rabbit anti-Mouse Polyclonal (aa26-44) Antibody LSBio Cat# LS-C735 - Saba Y. et al. PNAS (2022)
Rat PE/Cyanine7 anti-mouse/human CD45R/B220 BioLegend Cat# 103222; RRID: AB_ 313005 - Zubeidat K. et al. Cell Reports (2023)

Rat Brilliant Violet 711™ anti-mouse Ly-6C BioLegend Cat# 128037; RRID: AB_ 2562630 - Wald S. et al. JDR (2021)
Rat PE/Cyanine7 anti-mouse/human CD11b BioLegend Cat# 101216; RRID: AB_ 312799 - Wald S. et al. JDR (2021)
Rat APC/Cy7 anti-mouse CD4 BioLegend Cat# 100414; RRID: AB_ 312699 - Wald S. et al. JDR (2021)
Rat PE/Cyanine5 anti-mouse CD8a BioLegend Cat# 100709; RRID: AB_312749 - Zubeidat K. et al. Cell Reports (2023)
Rat Brilliant Violet 650TM anti-mouse/human CD11b BioLegend Cat# 101259; RRID: AB_ 2566568 - Zubeidat K. et al. Cell Reports (2023)
Rat PE/DazzleTM 594 anti-mouse Ly-6G BioLegend Cat# 127647; RRID: AB_ 2566318 - Zubeidat K. et al. Cell Reports (2023)
Armenian hamster APC/Cyanine7 anti-mouse CD11c BioLegend Cat# 117324; RRID: AB_ 830649 - Barel O. et al. JDR (2022)
Rat anti-Mouse I-A/I-E BUV496 M5/114.15.2 BD Biosciences Cat# 750281; RRID: AB_ 2874472 - Zubeidat K. et al. Cell Reports (2023)
Rat BD741261 BUV563 anti-mouse CD103 BD Biosciences Cat# 741261; RRID: AB_ 2870808 - Zubeidat K. et al. Cell Reports (2023)
Rat Alexa Fluor® 488 anti-mouse FOXP3 BioLegend Cat# 126406; RRID: AB_ 1089113 - Saba Y. et al. PNAS (2022)

## Animals and other research organisms

Policy information about studies involving animals; ARRIVE guidelines recommended for reporting animal research, and Sex and Gender in Research

| | |
|---|---|
| Laboratory animals | C57BL/6 (B6), langerin-DTR, B6.129P2-Il17 atm1Yiw (Il17a –/– ) and Germ-free (GF) B6 mice were involved in this study. The mice were euthanized at ages 1,2,4,6 or 8 weeks depending on the specific experiment. Housing conditions were 21-24°C, 30-70% humidity, with lights in the room for 12 hours, between 7:00-19:00, and dark for 12 hours from 19:00-7:00. |
| Wild animals | The study did not involve wild animals. |
| Reporting on sex | Finding apply to both sexes. sex matched mice were chosen in groups of each experiment. |
| Field-collected samples | The study did not involve field-collected samples. |
| Ethics oversight | All animal protocols were approved by the Hebrew University Institutional Animal Care and Use Committee (IACUC). GF studies were approved by the IACUC of the Weizmann Institute of Science. |

Note that full information on the approval of the study protocol must also be provided in the manuscript.

## Flow Cytometry

### Plots

Confirm that:

☒ The axis labels state the marker and fluorochrome used (e.g. CD4-FITC).

☒ The axis scales are clearly visible. Include numbers along axes only for bottom left plot of group (a 'group' is an analysis of identical markers).

☒ All plots are contour plots with outliers or pseudocolor plots.

☒ A numerical value for number of cells or percentage (with statistics) is provided.

### Methodology

| | |
|---|---|
| Sample preparation | The gingival tissues and lymph nodes were excised. In some of the experiments gingival tissues were incubated in 1mL of 4mg/ml Dispase in PBS + 2% FCS until fully distended for 30 minutes. The epithelium and sub-epithelium were carefully separated using forceps and binocular microscope. Tissues were then minced and treated with a Collagenase type II (2 mg/mL) and DNase I (1 mg/mL) solution in PBS plus 2% FCS for 25 min at 37 °C in a shaker bath. A total of 20 µL of 0.5 M EDTA per 2 mL sample was added to the digested tissues and incubated for an additional 10 min. The cells were then washed, filtered with 70-µM filter, incubated with antibodies for 20 minutes on ice in the dark and washed. |
| Instrument | Samples were ran in Aurora (Cytek) flow cytometer. |
| Software | Data was analyzed generating plots and tSNE plots using FlowJo software (BD Biosciences). |
| Cell population abundance | The frequency of Leukocytes was about 20% of all cells in the gingival mucosa. |
| Gating strategy | Singlets were gated according to the pattern of SSC-A vs. SSC-H and FSC-A vs. FSC-H. All cells were gated based on the pattern of FSC-A/SSC-A. Leukocytes were gated based on negative expression of autofluorescence and positive expression of CD45. Further expression pattern of MHCII and CD11b divided Leukocytes into B cells (MHCII+CDIIb-), T cells and ILCs (MHCII-CD11b-), myeloid cells (MHCII-CD11b+) and APCs (MHCII+CD11b+).<br>In some experiments, further gating on MHCII+CD11c+ Leukocytes identified APCs, that were further analyzed based on Langerin and EpCAM expression, marking Langerhans cells. In other experiments, gating on FITC out of APCs or Langerhans cells identified oral mucosa originating migrating cells.<br>In staining for T regulatory cells, Leukocytes were further gated for CD3 and CD4, and double positive cells were gated for FOXP3 and abTCR, marking the double positive population as T-reg cells. |

☒ Tick this box to confirm that a figure exemplifying the gating strategy is provided in the Supplementary Information.
